# Cervical cancer screening and treatment for PLWHIV: experiences from an innovative program in Nigeria

**DOI:** 10.1186/s12978-023-01658-0

**Published:** 2023-08-26

**Authors:** Olabanjo Okunlola Ogunsola, Oluseye Ayodele Ajayi, Temitope Olumuyiwa Ojo, Emmanuel Osayi, Kucheli Wudiri, Babatunde Amoo, Rita Ayoka-Ikechukwu, Olufemi Ojuola Olumeyan, Chukwuemeka Ifechelobi, Prosper Okonkwo, Yewande Akinro

**Affiliations:** 1 APIN Public Health Initiatives, Abuja, Nigeria; 2 Department of Community Health, Obafemi Awolowo University, Ile-Ife, Nigeria

**Keywords:** Cervical cancer screening, WLHIV, Nigeria

## Abstract

**Background:**

We evaluated cervical cancer program for women living with HIV (WLHIV) to determine program screening rate, primary case finder screening accuracy and treatment and post-treatment screening rate among screen-positive patients.

**Methods:**

A ten-month review of cervical cancer program data among WLHIV aged 15–49 years on HIV care across forty-one comprehensive ART sites, supported by APIN (a PEPFAR implementing partner) for cervical cancer screening and treatment in Nigeria, was conducted from October 2020 to July 2021. Initial screening was done using visual inspection with acetic acid (VIA) followed by a gynaecologist expert review through a program-designed software named AVIVA, as a confirmatory test. Associations were measured between the primary case finder screening accuracy and study covariates at p-value of 0.05.

**Results:**

About 10,289 asymptomatic women aged 15–49 years living with HIV were screened for cervical cancer by primary case finders using VIA-based screening test. About 732 (7.1%) had a positive screening test suggestive of precancerous lesions or cervical cancer. Three hundred and fifteen (43.0%) of VIA positive women had treatment using thermal ablation and less than one-third (21.6%) of those treated came back for post-treatment screening test. Primary case finder screening sensitivity, specificity, positive predictive and negative predictive accuracy using gynaecologist review as confirmatory test were 60.8%, 71.5%, 41.7% and 84.5% respectively. Overall screening accuracy was 68.8%.

**Conclusion and recommendations:**

This innovative approach to cervical cancer screening among WLHIV yielded modest results in preventing program error and wastages. Wider deployment of expert-based reviews of VIA though AVIVA software might be a veritable approach to improve screening accuracy in low resource settings.

## Background

Cervical cancer is the fourth most prevalent malignancy among women worldwide. In 2018, more than 500,000 women were diagnosed with cervical cancer, and nearly half of them succumbed to the disease [1]. Cervical cancer remains the primary cause of cancer-related deaths among women in countries with limited resources, particularly in Africa [1]. Compared to women without HIV, HIV-positive women have a sixfold increased risk of developing cervical cancer [2]. This increased risk manifests itself throughout the lifecycle, beginning with an increased risk of infection with human papillomavirus serotypes 16 and 18, an organism implicated in 70–75% of cervical cancer cases worldwide [2, 3]. In sub-Saharan Africa, HIV epidemics are the leading cause of cervical cancer [2].

In Nigeria, one of the sub-Saharan African nations with HIV epidemics, cervical cancer rates second among women of reproductive age, after breast cancer [4, 5]. As of 2019, 14,943 new cases of cervical cancer were diagnosed annually in women, with a case fatality rate of 70% [6] in the United States. With a prevalence of 66.9% among cervical cancer patients in Nigeria, HPV infection remains the most implicated causative agent for cervical cancer. Estimates of HPV infection among sexually active women in Nigerian states ranged from 76% in Kano and 48.1% in Gombe to a decreased prevalence of 19.6% and 26.3%, respectively, in Lagos and Oyo [7–9]. Among women living with HIV in Nigeria, the prevalence of high-risk HPV and cervical precancerous lesions is 19.6% and 6.0%, respectively, compared to their HIV-negative peers, with HPV 16 being the most prevalent serotype [8]. In 2013, the World Health Organization (WHO) launched the three-level prevention strategies for the control of cervical cancer, with an emphasis on HPV vaccination for children aged 9 to 13 years, early detection via screening for pre-cancerous changes in the cervix, and treatment of invasive cervical cancer [10]. Further, they proposed a 'screen and treat' approach to prevention for resource-poor contexts, employing the low-cost technologies of visual inspection with acetic acid (VIA) and cryotherapy. This was in accordance with the 2008 cancer control plan adopted by the Nigerian Federal Ministry of Health (FMOH), which recognized cervical cancer screening with visual inspection using acetic acid or Lugol’s iodine and cryotherapy treatment of precancerous lesions [11].

A screening test based on VIA has numerous advantages over other cervical cancer screening methods. It is cost effective, requires less expertise in conducting and interpreting, and is ideally suited for community-based clinics where examination privacy and a decent light source can be ensured. However, some studies [12–14] have shown that the reliability of VIA in detecting precancerous cervical lesions varies widely. A meta-analysis that pooled data from studies conducted among an asymptomatic population and used colposcopy and histologic testing as confirmatory methods reported high sensitivity and specificity for VIA-based tests, with a pooled sensitivity rate of 80% and a specificity rate of 92%, and no significant differences in testing accuracy in relation to the type of health care worker who conducted the screening [14]. Vahedpoor et al. [15] reported a higher sensitivity and specificity rate for VIA-based tests in India, as well as a reduced false negative rate of 4.6% and a higher false positive rate of 21.2% compared to Pap smears. Gravitt et al. [13] in India, on the other hand, reported a very low sensitivity rate for VIA-based assays compared to HPV testing and cytology. Compared to HPV testing (100%) and pap cytology (78.2%), VIA screening was only able to identify 31.6% of screened women with precancerous lesions using histology; however, VIA specificity remained high (87.5%) [13].

All these studies reported a low positive predictive value for VIA-based diagnostic assessments [13, 16–18]. A substantial proportion of women who test positive for VIA may not actually have the disease, resulting in misdiagnosis, excessive treatment, and unwarranted distress among those who are tested. Other major concerns with the VIA test include its subjectivity, which makes interpretation variable depending on the health care provider, the absence of a permanent test record for possible future review, and its limited reliability for screening postmenopausal women whose cervical regions have likely undergone transformation [19].

APIN introduced multiple levels of evaluation of VIA cervix-stained images using APIN-developed software called AVIVA to improve the accuracy of VIA-based screening tests and mitigate the social and economic repercussions of misdiagnosis resulting from the test's low positive predictive value. AVIVA is a lightweight Android mobile application built with Google's front-end programming language, Angular version 11, packaged for mobile deployment with Ionic, and operating on the Apache Cordova mobile application development framework. The framework some essential services that improve the VIA screening service for both case finders and specialist reviewers. It uses services such as the HTTP API (Application Programming Interface) that runs a real-time handshake with a cloud-based data warehouse system for the storage of all captured images and analytics to determine the level of concurrence between case finder diagnosis and reviewer diagnosis, track the progress of the case finder in closing the concurrence gap, and display performance at the case finder, facility, state, and program levels, respectively. Using the application and SMS, a notification service for real-time alerts and a feedback mechanism for case finders when their findings have been assessed are implemented.

The case finder makes the first diagnosis via tying up the findings from a direct visual inspection of the native, acetic and Lugol’s iodine-stained cervix respectively. While making the baseline diagnosis, she takes serial, clear and well-illuminated three images of the native cervix, acetic and Lugol’s iodine-stained cervix respectively and uploads on the AVIVA. Mainly positive VIA test results or cases in doubt is expected to be uploaded on the AVIVA app. Then designated gynecologists, who also have access to the pictures, is prompted by a notification sound that beeps every 5 min to provide expert review. Same notification is sent to the APIN State and central cervical cancer focal persons who also call the attention of the expert reviewer on the uploaded cases. On review, the expert reviewer proffers the necessary treatment modalities based on observed findings. The case finder within minutes receives the feedbacks and adequately attend to the patient still on the couch.

This study aimed at estimating (1) the prevalence and treatment outcome of precancerous lesion in APIN cervical cancer screening and treatment program, (2) the case finder screening sensitivity, specificity, positive and negative predictive value, (3) factors associated with case finder screening accuracy, and (4) the case finder screening error rate that could be prevented by using sequential expert gynaecologist agreement as a confirmatory test before treatment.

### Methods

#### Study design and setting

The study conducted a review of cervical cancer screening and treatment program data of women of reproductive age living with HIV in 41 comprehensive ART sites (3 Primary Health Facilities, 25 Secondary Health Facilities, 13 Tertiary Health Facilities) in 7 APIN supported states in Nigeria (Benue, Plateau, Ondo, Ekiti, Oyo, Osun and Ogun states), within a ten-month period. The 41 sites were the sites supported by APIN for cervical cancer screening and treatment services between 2020 to 2021. APIN is one of PEPFAR funded non-governmental organizations in Nigeria responsible for the provision of prevention, care and treatment services to patients with HIV/AIDS. APIN has data for more than 1 million patients with over 340,000 currently enrolled in her ART care program in eight states and in over 4400 supported health facilities. A comprehensive site in APIN program is either a secondary level or tertiary level health facility. These facilities serve as a referral center for provision of secondary or tertiary care for most primary and secondary level health facilities. The HIV clinic in the comprehensive sites provides ART services to paediatric, non-pregnant adult patients and PMTCT services to pregnant ART patients. It has different sub-units including adherence counselling unit, consulting rooms, records, phlebotomy unit, patient waiting room and data room for electronic medical records (EMR) operations. Most of the facilities open every weekday and operate block appointment for cohort of patients with similar attributes.

### Procedure

**Fig. 1:**
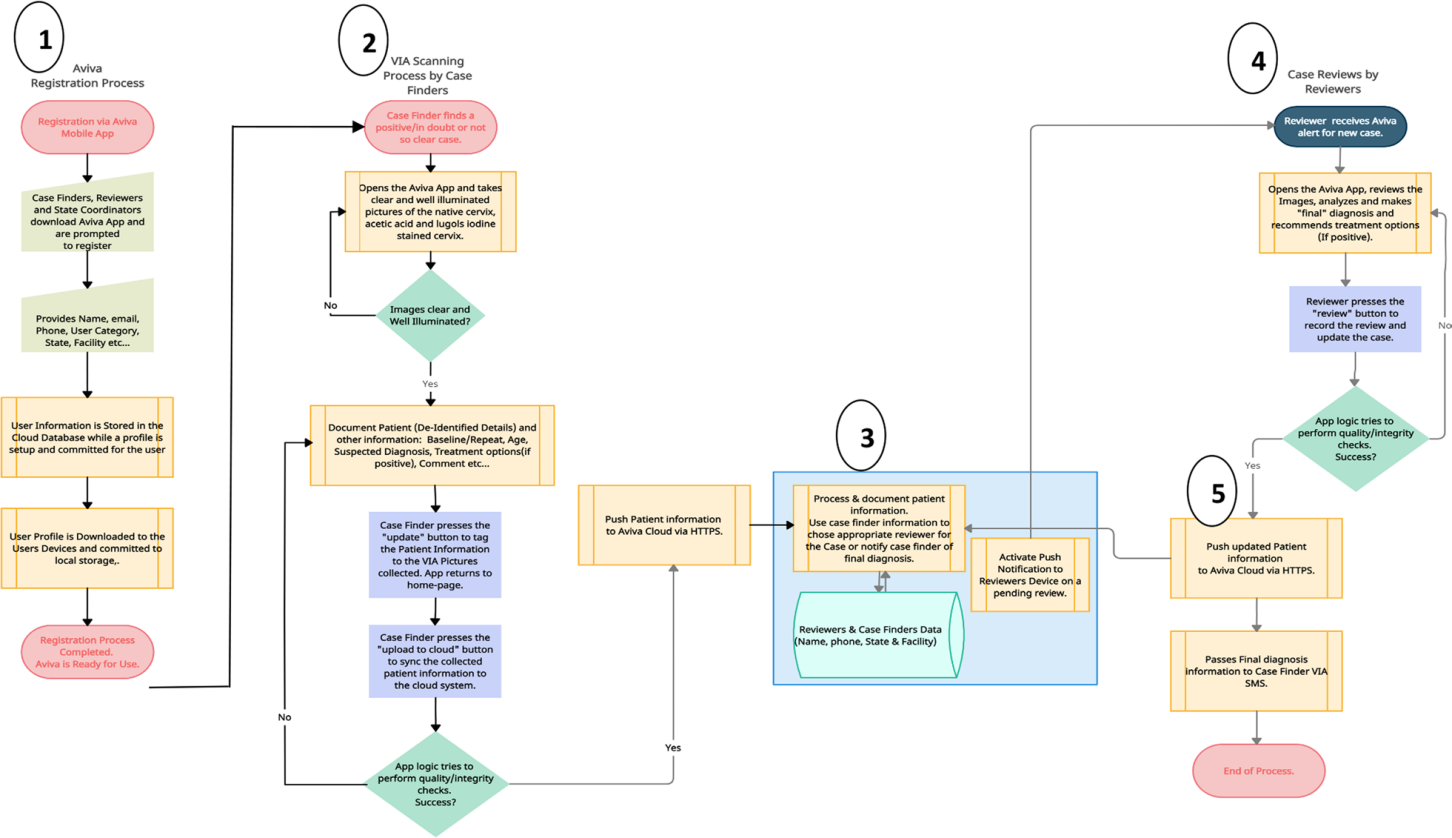
The flowchart of cervical cancer screening using the AVIVA software to facilitate the process

Furthermore, in assessing cervical cancer screening accuracy, a ten-month review of the piloted electronic VIA cervical images of women who received HIV care from 16 selected comprehensive care sites) was conducted across three administrative regions in Nigeria; Plateau region, Oyo region (Osun and Oyo states) and Ondo region (Ondo and Ekiti states). These images were taken by primary case finders (community health extension workers and Nurses) across APIN Public Health Initiatives (APIN) supported health facilities, uploaded to a software (named AVIVA) developed by the organization and were subsequently reviewed by corresponding expert gynaecologists. One primary case finder each was selected from the sixteen comprehensive sites for HIV care in the administrative regions. The selected comprehensive sites comprised of nine secondary health facilities (SHFs) and six tertiary health facilities (THFs). The primary case finders in this study were mandated to conduct cervical cancer screening for all consenting women living with HIV aged at least 15 years and enrolled in ART care, using the visual inspection with acetic acid (VIA) screening method, and upload all positive VIA cases and 10% of all negative VIA cases to the APIN AVIVA software for expert gynaecologists review and concurrence. A total of seven trained gynaecologists were engaged on the project, one per state, to do daily review of case finder’s uploads and post comments in agreement or disagreement with case finder diagnosis. Following agreement of positive results of precancerous lesion by reviewer, the cases are scheduled for immediate thermal ablation and 6 months post treatment screening to confirm treatment success in them. The details of this process are included in Fig. 1

A total of 295 cervical visual images were uploaded by primary case finders for gynaecologist review over the ten-month period; 187 negative images and 108 positive images. Positive VIA test result was defined as the presence of acetowhite lesions in the transformation zone, near the squamocolumnar junction or the os of the cervix, one minute after the direct application of a 3% to 5% diluted solution of acetic acid, while absence of this is termed as negative result.

### Study variables

**Fig. 2:**
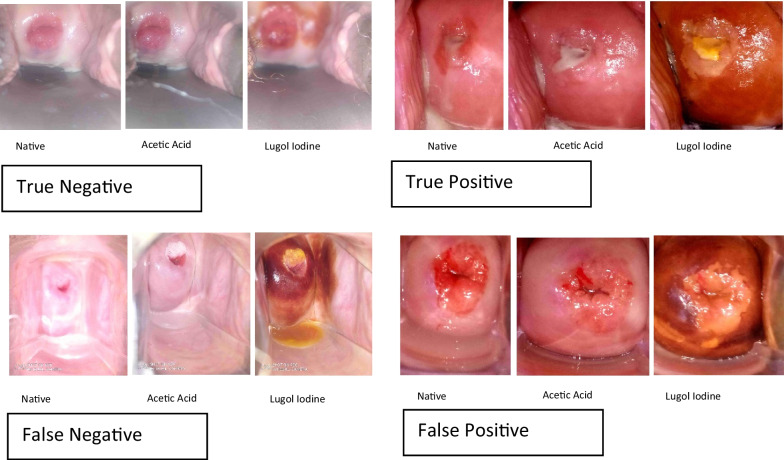
Visual images of various categories of screening outcomes

The primary case finder assessment is considered as the primary screening test while the AVIVA reviewers’ comments are considered as a confirmatory test. The numbers of each combination of results—true positive (TP; positive results to both VIA and confirmatory testing), false positive (FP; positive VIA result but negative result to confirmatory testing), false negative (FN; negative VIA result but positive result to confirmatory testing), and true negative (TN; negative result to both VIA and confirmatory testing) were extracted from the study, and the following 4 parameters of accuracy and performance for VIA were estimated: sensitivity = $(TP/TP+FN)*100$ , specificity = $(TN/TN+FP)*100$, positive predictive value PPV = $TP/TP+FP$, and negative predictive value NPV = $TN/TN+FN.$ See Fig. 2 for images of true positives, false positive, true negative and false negative.

The outcome indicator of this study was case finder VIA report accuracy rate and this was estimated as $[TP+TN/TP+FP+TN+FN]*100$.

### Data analysis

Data were entered into an Excel spreadsheet (Microsoft), cleaned and analysed using SPSS IBM version 20. Data were presented using tables and charts. T-test analysis was utilized to examine association between covariates and case finder cervical VIA screening accuracy rate. Covariates examined were region, state, facility level of care.

### Ethical approval

Ethical approval was obtained from APIN research institution board.

## Results

Ten thousand, two hundred and fifty-nine (10,259) asymptomatic women aged 15–49 years living with HIV were screened for cervical cancer by primary case finders across 41 health facilities in 7 APIN supported states in Nigeria, using VIA based screening test from October 2020 to July 2021. About 732 (7.1%) had a positive screening test suggestive of a precancerous lesion or cervical cancer. More women were positive in North-Central region (7.7%), Plateau state (15.8%) and tertiary health facilities (11.7%), when compared to South-Western region, other states and level of health care (Table 1).

**Table 1:** Primary case finder cervical cancer screening outcome by facility characteristics

Variable (s)	Case finder VIA screening test outcome		Total
	Positive (*n* = 732)	Negative (9527)	
	Freq (%)	Freq (%)	
*Region*			
North Central	565 (7.7)	6764 (92.3)	7329
South West	167 (5.7)	2763 (94.3)	2930
*State*			
Benue	150 (3.2)	4558 (96.8)	4708
Plateau	415 (15.8)	2206 (84.2)	2621
Ekiti	4 (1.3)	307 (98.7)	311
Oyo	123 (9.3)	1203 (90.7)	1326
Osun	2 (0.7)	299 (99.3)	301
Ondo	38 (8.7)	400 (91.3)	438
Ogun	0 (0.0)	554 (100.0)	0
*Level of care*			
Primary	0 (0.0)	41 (100.0)	41
Secondary	239 (4.0)	5751 (96.0)	5990
Tertiary	493 (11.7)	3735 (88.3)	4228

The overall treatment rate and retention using thermal ablation therapy across the states were below average as only 315 (43.0%) patients identified with precancerous lesion had thermal ablation therapy and less than one-third (21.6%) of those who had treatment returned for post treatment screening (Table 2).

**Table 2:** Summary of treatment history for positive VIA clients

PLHIV who had positive test outcome	VIA positive patients treated who had thermal ablation therapy	Treated patients who returned for 6 months post treatment screening
Number	Freq (%)	Freq (%)
732	315 (43.0)	68 (21.6)

Furthermore, two hundred and ninety-seven (297) cervical visual images stained with acetic acid (VIA reports) of women living with HIV age 15 years and above in 16 health facilities across the 5 APIN supported states were uploaded for expert gynaecologists’ review using AVIVA software from October 2020 to July 2021. Majority of the uploads were from North Central Region, 215 (72.9%), Plateau states 215 (72.9%), and SHFs, 269 (91.2%) (Table 3).

**Table 3:** Background characteristics of VIA images uploaded

Variable (s)	Frequency	Percent
*Region*		
North Central	215	72.9
South West	80	27.1
Total	295	100.0
*APIN Administrative regions*		
Plateau Region	215	72.9
Oyo Region (Osun and Oyo)	69	23.4
Ondo Region (Ondo and Ekiti)	11	3.7
Total	295	100.0
*Level of care*		
Secondary	269	91.2
Tertiary	26	8.8
Total	295	100.0

Forty-five out of the 108 positive cases identified by case finders VIA test were truly positive, resulting in a positive predictive rate (PPV) of 41.7%, while 158 out of 187 negative cases identified by case finders were truly negative, giving a negative predictive value (NPV) of 84.5%. The case finder VIA screening test sensitivity and specificity rates were 60.8% and 71.5% respectively. The expert review through AVIVA software was able to prevent type 1 error (False positivity error) among 63 positives (28.5%) and type 2 error (False negative error) among 29 negatives (39.1%) (Table 4).

**Table 4:** Comparison of case finder screening to AVIVA expert reviewers’ confirmation

Variable (s)	AVIVA reviewer's report			Predictive value (%)
	Negative	Positive	Total	
	Freq (%)	Freq (%)		
*Case finder CIN screening results using VIA*				
Negative	158 (84.5)	29 (15.5)	187	NPV = 84.5
Positive	63 (58.3)	45 (41.7)	108	PPV = 41.7
Total	221 (74.9)	74 (25.1)	295	
Accuracy (%)	Specificity = 71.5	Sensitivity = 60.8		

Overall accuracy of case finder screening test = TN + TP/Overall total = 68.8%

Case finders in the north central region had higher overall mean screening test accuracy rate (74.8) compared to those in South west region (27.4) (p = 0.03). Similarly, case finders in Plateau (74.8) and Ondo state (70.8) had higher mean screening test accuracy rate compared to those in Oyo state (17.7) (p = 0.01) (Table 5).

**Table 5:** Factors associated with the accuracy of case finder VIA screening

Variable (s)	*N*	Mean	Std. deviation	*F* or *t*-statistics	*df*	*P* value
Geographical Region						
North Central	11	27.4	33.7	− 2.51	14	0.03*
South West	5	74.8	38.2			
Facility level of care						
Secondary	11	42.2	44.1	< 0.01	14	1.00
Tertiary	5	42.1	37.0			
APIN administrative region						
Plateau	5	74.8	38.2	6.37	2	0.01*
Oyo	9	17.7	29.0			
Ondo	2	70.8	5.9			

## Discussion

Our study shows a prevalence of 7.1% precancerous or cervical cancer lesions among WLHIV, approximately 710 cases per 10,000 population. Our finding compares with a previous study conducted in Nigeria Federal Capital Territory, where prevalence of precancer or cervical cancer lesions was estimated as 6.0% using visual inspection with acetic acid (VIA) screening test [21]. However, it was far lower than prevalence of cervical intraepithelial lesions reported by two other studies done in Enugu and Jos, where estimated prevalences were 12.6% and 29.0% respectively. Of note was that these two studies adopted cytology-based testing approach which is a more effective, albeit expensive, screening test [20, 21]. Many studies also reported higher prevalence of cervical precancerous lesion among HIV positive women compared with HIV negative women in Nigeria.

Our study also showed low retention along the cervical cancer continuum of care. Overall, out of 732 WLHIV diagnosed with precancerous lesions 43.0% (315) had thermal ablation therapy while less than 40% came back for post treatment screening. Reasons for poor adherence to treatment and screening schedules remain unknown consequently, this requires further investigation but could include additional cost of transportation to facilities for retesting [22]

In this study, we also reported geographical variation in cervical cancer among WLHIV. This suggests a much higher risk of developing cervical cancer in north central Nigeria compared to south west part of the country. Although 72.9% of the samples uploaded were cases from North central part of Nigeria which may explain why there was a higher positive screening test result from North Central Nigeria. Nevertheless, understanding the risk factors and reasons behind geographical disparities is critical to defining priority areas for cervical cancer prevention efforts and targeting women who are most likely to be at risk. Our findings also compares with similar studies reported by Marie-Josèphe et al. [23], where geographical and racial/ethnic variation were identified and reported in cervical cancer incidence and mortality in United States [23].

The study went further to examine the predictive ability and accuracy of the primary case finder VIA imaging using APIN’s developed AVIVA technology as confirmatory test. Sensitivity, specificity, positive and negative predictive values were calculated to be 60.8, 71.5, 41.7 and 84.5 respectively. The confirmatory test suggests that 15% of WLHIV who received VIA negative results could have received a false-negative screening result if they were tested alone with VIA. Conversely, 58.3% of WLHIV who received VIA positive results could have received a false-positive screening result if tested alone with VIA while 41.7% tested positive to both VIA and AVIVA confirmatory testing. Other studies that have adopted VIA screening tests reported similar findings regarding the low sensitivity and PPV but higher specificity and NPV of VIA test results [24, 25].

Another significant finding in our study revealed 58.3% false positives following AVIVA confirmatory tests demonstrating the need for further testing to confirm the presence or absence of cervical cancer. These false positives possibly could be attributable to confounding factors or errors during testing and interpretation by primary case finders during VIA screening tests. However, APIN’s AVIVA technology was critical in avoiding over-diagnosis furthermore, such outcome (false positives) following confirmatory test using AVIVA could pave new ways to improve performance and accuracy of VIA based screening program at all APIN’s cervical screening facilities.

In this study, we observed comparable case finder VIA screening accuracy among case finders in secondary health facilities when compared to their counterparts in tertiary facilities. Although the case finder screening accuracy was observed to be low (< 43%) across both levels of healthcare, this finding suggests that VIA screening can be performed by competent healthcare providers at all levels of healthcare when properly equipped with necessary training. VIA test is recommended for use in low resource settings like our study location [26, 27]. In Nigeria, secondary and tertiary health facilities may not be easily accessible to patients eligible for specific healthcare services [28], including HPV screening test services. Thus, availability of competent HPV case finders across all levels of healthcare is necessary in addressing cervical cancer control in the country. Although, there is also a need to ensure second level review of screening test results in these facilities, considering the low PPV of VIA test results observed in this study. Studies have shown comparable accuracy in the screening test results among different cadres of healthcare workers [29, 30].

According to various studies conducted in Nigeria, evidence exists that the prevalence of HPV infection is higher in the northern part of the country when compared to the southern region of the country [7, 31–33]. The prevalence of a disease condition affects the diagnostic accuracy of screening tests [34]. The lower VIA test accuracy observed among case finders in the southern region compared to their northern counterparts could be influenced by the lower prevalence of HPV infection in the region. The knowledge of the prevalence of a disease condition could influence reader expectation of a screening test result and hence, its accuracy [34].

A major limitation of the study is the use of only one expert reviewer to review VIA images for each screening test. Generally, the subjective nature of VIA affects its validity as interpretation of test results could be different from one health care provider to another [19].

Future studies to assess the yield and reliability of VIA in relation to the social and biological characteristics of WLHIV are appropriate. Also, implementation science research using some innovative approaches in improving the reliability of VIA is required. Such approaches may entail randomised clinical trials which will employ a combination of colposcopy, cytology, histology and expert review of VIA images by histopathologists among others. In addition, longitudinal studies with validation cohorts are required to obtain relevant data needed for net benefit analysis and bootstrap modelling for multiple outcomes.

## Conclusion and recommendations

In conclusion, the innovative approach to cervical cancer screening (AVIVA) adopted by APIN in its cervical cancer control program among WLHIV appears to have yielded modest results in terms of its reliability and feasibility. The “AVIVA model” may be a veritable tool for cervical screening in low resource settings if the health workers (case finders) who perform visual inspection with Acetic acid can be further trained to ensure optimal standards. In addition, the integration of cervical cancer screening program into existing ART programs as demonstrated in this study is feasible, practicable and realistic and this is recommended as a standard of care for all eligible WLHIV who are accessing care at any comprehensive ART site.

There is the need to establish a cervical cancer awareness and education program for WLHIV as part of comprehensive ART services, so that they are readily motivated to uptake cancer screening services as required. Also, a special counselling and follow up program is recommended for persons who get screened for cervical cancer to reduce loss to follow and default from treatment for precancerous lesions. We recommend that an appropriate policy framework for cervical cancer screening as part of routine care for WLHIV should be provided to ensure that such screening programs are institutionalized and sustainable. Our involvement of key stakeholders, including policy makers, healthcare workers, patients and implementing partners will contribute to the expansion and sustainability of AVIVA’s usage. Furthermore, the use of AVIVA, which has helped to reduce misdiagnosis of acetic-acid-stained cervix, could enable health facilities in resource-limited settings to provide quality cervical cancer screening services. In settings like ours, where HPV DNA testing and pap smears are not widely available and out of reach for poor and rural populations, VIA testing with AVIVA's support will assure access to and quality of services.

## Data Availability

The survey data is available upon reasonable request.
